# Supplementary material for: Patients’ experiences of a Virtual Fracture Assessment Clinic Pathway: A qualitative study
Source: PLoS One. 2025 Apr 7;20(4):e0321400. doi: 10.1371/journal.pone.0321400 (PMC11975123; doi:10.1371/journal.pone.0321400)
Supplement: S2 Table — (PDF) [file pone.0321400.s002.pdf]

S2 Table. Purposive Recruitment Sample in Regard to Patients Referred to the vFAC Pathway in 2023.

		Purposive sample from current study	Patients referred to the vFAC pathway in 2023
**Sex:**	Male	58%	54%
	Female	42%	46%
**Age Range:**	16-18 Years Old	0%	2%
	18-24 Years Old	17%	13%
	25-34 Years Old	33%	22%
	35-44 Years Old	0%	17%
	45-54 Years Old	8%	15%
	55-64 Years Old	8%	13%
	65+ Years Old	33%	17%
**Geographic Location:**	Urban	75%	74%
	City	25%	21%
	Rural	0%	5%

**Presented to:**	IU	92%	93%
	ED	8%	7%
**Injury:**	Shoulder	7%	13%
	Elbow	14%	9%
	Wrist	21%	23%
	Hand	7%	25%
	Hip	0%	1%
	Knee	0%	4%
	Ankle	7%	10%
	Foot	43%	15%
**vFAC Management Plan:**	In-Person Fracture Clinic	33%	44%
	Physiotherapy	33%	20%
	Discharge	33%	30%

	Occupational Therapy	0%	1%
	Surgery	0%	1%
	Referred back to ED	0%	2%
	Other	0%	2%
