# Supplementary material for: Patients’ experiences of a Virtual Fracture Assessment Clinic Pathway: A qualitative study
Source: PLoS One. 2025 Apr 7;20(4):e0321400. doi: 10.1371/journal.pone.0321400 (PMC11975123; doi:10.1371/journal.pone.0321400)
Supplement: S3 Table — (PDF) [file pone.0321400.s003.pdf]

S3 Table. Interview Guide.

**Introduction**	• Thank participant for their time<br>• Explain study aim and objectives<br>• Advise participant cannot offer advice on their injury, as not involved in their care, but can signpost to the appropriate person to contact<br>• Advise participant they are free not to answer any questions if they wish<br>• Advise on distress protocol<br>• Advise interview will be recorded<br>• Explain transcript anonymisation process<br>• Explain member checking process<br>• Ask participant if they have any questions

**Questions**	**Prompts**
1. Can you tell me about your injury?	• What injury did you sustain?<br>• Mechanism of injury
2. Can you clarify some background details about yourself?	• Past medical history<br>• Previous fractures<br>• Any previous experience of an in-person fracture clinic or vFAC pathway?<br>• Occupation<br>• Sports and hobbies<br>• Age range:<br>(18-24, 25-34, 35-44, 45-54, 55-64, 65+)<br>• Geographical location: city, urban or rural<br>• Family/Friend support network
3. Can you tell me about your experience of the IU/ED consultation?	• Did you attend the IU or the ED?<br>• What were your expectations for this phase?<br>• Were you treated by a doctor or an advanced nurse practitioner?

	• X-rays or other investigations (did you see your x-ray/scan?)<br>• Immobilisation device (advice on how to apply, when to wear or when to wean from device)<br>• Were you given crutches and advised on how to use these? (if applicable)<br>• Advice on managing pain and oedema<br>• Advice on weightbearing status (if applicable)<br>• Advice on expected recovery timeline<br>• Advice on activities of daily living<br>• Advice on work/hobbies (if applicable)<br>• Advice on driving (if applicable)<br>• Advice on exercises or rehab<br>• Advice on complications to be aware of<br>• Details provided on who to contact if any issues<br>• Explanation of the follow-up process<br>• What were your thoughts on being referred to the vFAC?

	• Opportunity to ask questions?<br>• Did you have any concerns at this stage?<br>• How did you find this phase from a psychological perspective?<br>• What advice would you like to receive in this phase?<br>• Written information given<br>• How would you like to receive information; paper, email, website, app, video recordings<br>• Positives and negatives of this phase<br>• How would you recommend improving this phase of the pathway?<br>• Anything else you would like to mention about this phase?
4. Can you tell me about your experience of the vFAC consultation?	• What were your expectations for this phase?<br>• Time interval between ED/IU and vFAC consultations?<br>• Was a phone consultation convenient for you?<br>• Were you referred back to the IU/ED for any further investigations?<br>• Plan for follow-up

	• Immobilisation device (advice on how to apply, when to wear or when to wean from device)<br>• Advice on crutches (advice on weaning from same? If applicable)<br>• Advice on managing pain and oedema<br>• Advice on weightbearing status (if applicable)<br>• Advice on expected recovery timeline<br>• Advice on activities of daily living<br>• Advice on work/hobbies (if applicable)<br>• Advice on driving (if applicable)<br>• Advice on exercises or rehab<br>• Advice on complications to be aware of<br>• Details provided on who to contact if any issues<br>• Explanation of the follow-up process<br>• Opportunity to ask questions?<br>• Did you have any concerns at this stage?

	• Did you have any concerns about lack of a physical assessment?<br>• Did you have any concerns about lack of repeat x-ray or other investigations? (if applicable)<br>• Did you have any concerns about possibility of misdiagnosis?<br>• Any conflicting advice compared to advice provided at the IU/ED?<br>• Preference for vFAC versus in-person fracture clinic consultation<br>• How did you find this phase from a psychological perspective?<br>• What advice would you like to receive in this phase?<br>• Positives and negatives of this phase<br>• How would you recommend improving this phase of the pathway?<br>• Anything else you would like to mention about this phase?
5. Can you tell me about your experience of the in-person fracture clinic consultation? (if applicable)	• What were your expectations for this phase?<br>• Did you expect to be followed up at the in-person fracture clinic after the vFAC consultation?<br>• Time interval between vFAC and in-person fracture clinic consultations?

	• Were you concerned you were being brought into an in-person fracture clinic?<br>• Were you relieved you were being brought into an in-person fracture clinic?<br>• Thoughts on having a repeat x-ray or other investigation (did you see your x-ray/scan?) (if applicable)<br>• Immobilisation device (advice on how to apply, when to wear or when to wean from device)<br>• Advice on crutches (advice on weaning from same? If applicable)<br>• Advice on managing pain and oedema<br>• Advice on weightbearing status (if applicable)<br>• Advice on expected recovery timeline<br>• Advice on activities of daily living<br>• Advice on work/hobbies (if applicable)<br>• Advice on driving (if applicable)<br>• Did you have a physiotherapy consultation at the in-person fracture clinic?<br>• Advice on exercises or rehab

	• Advice on complications to be aware of<br>• Details provided on who to contact if any issues<br>• Explanation of the follow-up process<br>• Opportunity to ask questions?<br>• Did you have any concerns at this stage?<br>• Any conflicting advice compared to advice provided at the IU/ED or vFAC consultations?<br>• Would you prefer to have been managed solely by the vFAC if possible?<br>• Positives and negatives of this phase<br>• How would you recommend improving this phase of the pathway?<br>• Anything else you would like to mention from this phase?
6. Can you tell me about your experience of the recovery/rehabilitation process?	• What were your expectations for this phase?

	<u>Physiotherapy consultation following in-person fracture clinic consultation:</u><br>• Time interval between in-person fracture clinic and physiotherapy consultations?<br>• Opportunity to ask questions?<br>• Any conflicting advice compared to advice provided at the IU/ED, vFAC or in-person fracture clinic consultations?<br>• Preference for self-management or physiotherapy consultation?<br><br><u>Discharged to physiotherapy following vFAC consultation:</u><br>• Time interval between vFAC and physiotherapy consultations?<br>• Thoughts on only having a physiotherapy consultation and not an in-person fracture clinic consultant.<br>• Preference for in-person fracture clinic consultation and then a physiotherapy consultation?<br>• Reassuring seeing a physiotherapist?<br>• Opportunity to ask questions?

	• Any conflicting advice compared to advice provided at IU/ED or vFAC consultations?<br>• Preference for self-management or physiotherapy consultation?<br><br><u>Discharged from vFAC with no further follow-up:</u><br>• Thoughts on self-management<br>• Did you have any concerns about not having a physiotherapy consultation?<br>• Preference for physiotherapy consultation?<br>• Were you given any exercises or advice for self-management<br><br><u>Questions for all participants:</u><br>• Experience of weaning from immobilisation device or crutches (if applicable)<br>• Thoughts on your recovery (pain, oedema, reduced movement and strength)<br>• Experience of weightbearing (if applicable)<br>• Was your recovery in line with the timeline provided? (if applicable)

	• Was the recovery timeline as you would have expected?<br>• Experience of returning to activities of activities of daily living<br>• Experience of returning to work/hobbies (if applicable)<br>• Experience of returning to driving (if applicable)<br>• Did you experience any complications?<br>• Did you contact the ED/IU, vFAC, in-person fracture clinic or physiotherapist or consider contacting them for further support?<br>• Did you need to contact another health care professional (i.e. GP or private physiotherapist) in relation to this injury?<br>• Do you have concerns in this phase?<br>• Do you have any concerns regarding your long term prognosis?<br>• How did you find this phase from a psychological perspective?<br>• What advice would you like to receive in this phase?<br>• Did you receive any advice from friends and family?<br>• Positives and negatives of this phase

	• How would you recommend improving this phase of the pathway?<br>• Anything else you would like to mention from this phase?
7. Is there anything else about your experience we haven't covered that you feel is important or you would like to discuss?	
