# Supplementary figures and images for: Patients’ experiences of a Virtual Fracture Assessment Clinic Pathway: A qualitative study
Source: PLoS One. 2025 Apr 7;20(4):e0321400. doi: 10.1371/journal.pone.0321400 (PMC11975123; doi:10.1371/journal.pone.0321400)

**S4 File. Participant Recruitment Process.**

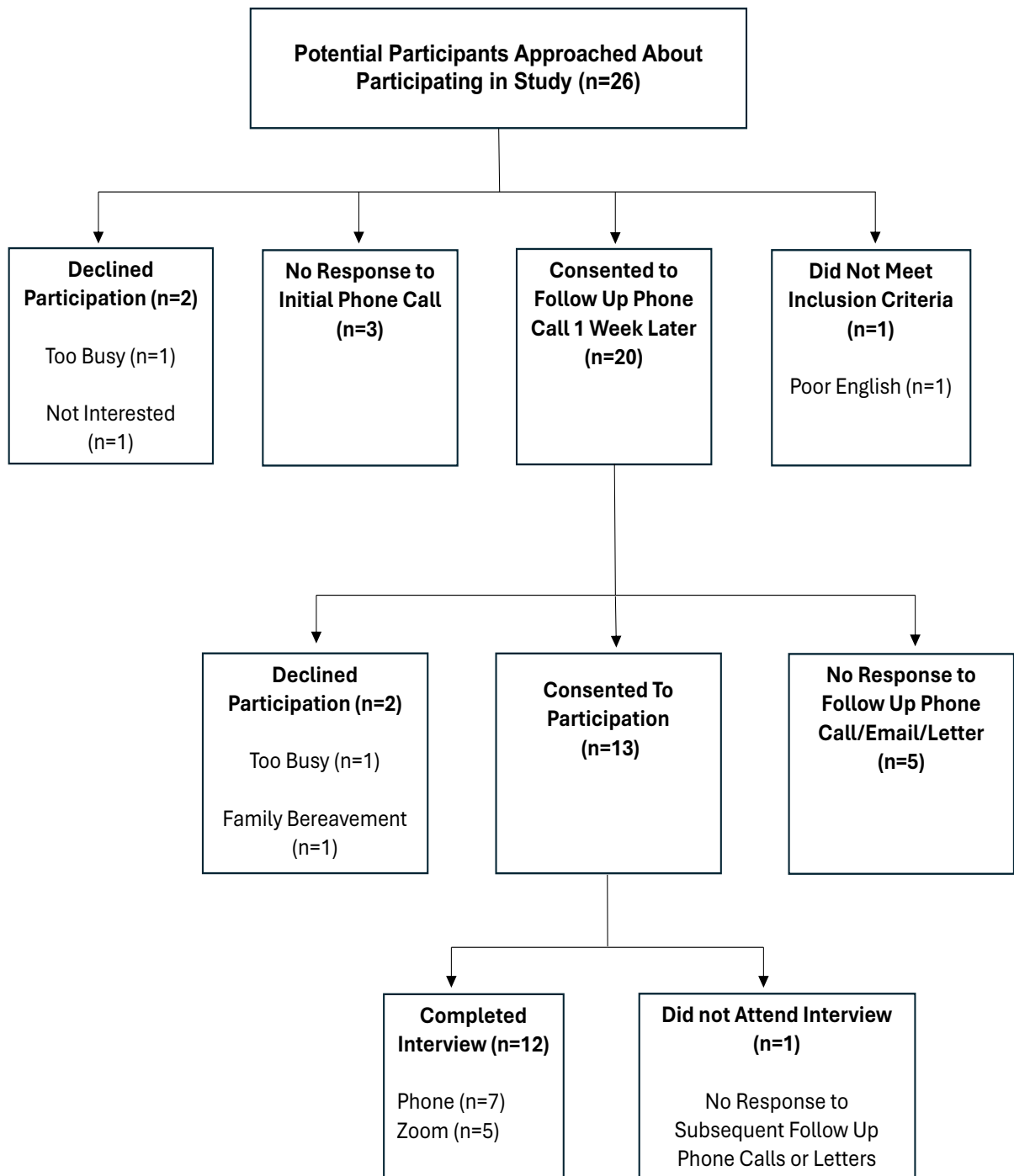

Supplement: S4 File — (PDF) [file pone.0321400.s004.pdf]
