# Supplementary material for: Patients’ experiences of a Virtual Fracture Assessment Clinic Pathway: A qualitative study
Source: PLoS One. 2025 Apr 7;20(4):e0321400. doi: 10.1371/journal.pone.0321400 (PMC11975123; doi:10.1371/journal.pone.0321400)
Supplement: Attachment — Submitted filename: Response to Reviewers.docx [file pone.0321400.s005.docx]

Darragh Carolan

Physiotherapy Department

Mater Misericordiae University Hospital

Eccles Street

Dublin 7

D07 R2WY

Ireland

darraghcarolan@mater.ie

01/02/2025

**Study Title: Patients’ experiences of a Virtual Fracture Assessment Clinic Pathway: A qualitative study**

Dear Dr. Zadro,

Many thanks for taking the time to review our submission to PLOS One. I have uploaded a marked up version of a revised manuscript and an unmarked version as requested. Please see below our response to points made by yourself and the 2 peer reviewers.

**Response to points from academic editor:**

***Improve the resolution of Fig1:***

The resolution of Fig1 has been improved using the recommended PACE digital diagnostic tool and uploaded to the editorial manager.

***Please include a complete copy of PLOS’ questionnaire on inclusivity in global research in your revised manuscript:***

Included (S1 Checklist. Inclusivity in Global Research Questionnaire Checklist.pdf).

***We note that you have indicated that there are restrictions to data sharing for this study. For studies involving human research participant data or other sensitive data, we encourage authors to share de-identified or anonymized data. However, when data cannot be publicly shared for ethical reasons, we allow authors to make their data sets available upon request:***

All relevant data are within the manuscript and supplementary information files. Relevant anonymised quotes from participants have been included in the results section of the manuscript. As per ethical approval obtained from the Mater Misericordiae University Hospital Institutional Review Board (reference number: 1/378/2387) and Manchester Metropolitan University Faculty Ethics Committee (reference number: 58689), anonymised versions of interview transcripts are saved on the Mater Misericordiae University Hospital computer system. Full anonymised transcripts can be made available on request. Requests can be sent to the Hannah King, Administrator, Institutional Review Board, Mater Misericordiae University Hospital, Eccles Street, Dublin 7, D07 R2WY, Ireland, [hannahking@mater.ie](mailto:hannahking@mater.ie), +353 18032971.

**Response to points from reviewer #1:**

All grammatical errors have been corrected as suggested (see revised manuscript with track changes).

**1. Consider changing ‘peripheral fractures’ to ‘peripheral limb fractures’ for improved clarity:**

Changed as suggested (see revised manuscript with track changes).

**2. In your Information section on page 15, you mentioned that participants highlighted the need for basic information on immobilisation devices. Did participants also mentioned the need for other information in the handouts such as their diagnosis, activity restrictions during the recovery period, and prognosis of recovery, or signs/symptoms to monitor e.g. deep vein thrombosis or complex regional pain syndrome?**

Participants only highlighted the need for more information about their diagnosis (due to receiving conflicting diagnoses at times) and using immobilisation devices, as mentioned in the results section. They did not mention a desire for information on diagnosis, activity restrictions, prognosis or signs/symptoms to monitor, perhaps as they felt this was adequately explained on the pathway.

**3. Regarding fragility fractures on page 24, you could consider adding this information into the patient’s standardised injury/recovery handout so that they can initiate the conversation with their usual health practitioner on screening for osteoporosis?**

This point has been added to the revised manuscript page 25, lines 535-537:

*‘Including information on fragility fractures in standardised patient information leaflets may encourage patients to discuss bone health screening with their general practitioner.’*

**4. In your results, you may want to add the mean (range) time of your interviews to provide readers a gauge on how in-depth the interviews were.**

Results section updated to include range of interview duration (30-75 minutes) and mean interview time (approximately 60 minutes). Page 12, lines 261-262.

**5. The addition of the S1 table is clear and reflects the closeness of your study participants’ demographics with the usual patients who utilise vFAC. Well done.**

No action required

**6. Thank you for sharing S3 Table – Distress Protocol. I’m not sure how important, or how this adds to this publication. You can consider removing this.**

Distress protocol removed from supplementary information.

**7. Finally, was there a general preference on which pathway patients preferred? I.e. did patients who were seen by a physiotherapist ‘happier’ than those who were ‘discharged’ vs those who were seen at the in-person clinic by a surgeon?**

There was no general preference on which pathway patients preferred. As mentioned in the results section, each participant had different preferences, i.e. some were happy to be referred to physiotherapy, while other were happy to self-manage their recovery. Similarly, some participants preferred to convenience of a virtual fracture clinic consultation, while others preferred attending an in-person fracture clinic.

**Response to points from reviewer #2:**

**Interviewed patients are similarly matched to the cohort of patients who received vFAC care in 2023, although there are interviews lacking from the 35-44yo cohort (0% vs 17%):**

This is a fair critique and a weakness in the sample recruited.

**I am unable to view figure 1. (thematic map). I am not sure if this is a paper issue or user error.**

The resolution of Fig1 has been improved using the recommended PACE digital diagnostic tool and uploaded to the editorial manager.

**Two minor spelling considerations:**

All spelling errors have been corrected as suggested (see revised manuscript with track changes).

**Additional points to note from the research team:**

1. On the editorial manager when selecting ‘view publication fee’ it states ‘your institution, University College Dublin, has a publication agreement with PLOS and will pay all or part of your publishing costs’. The Mater Misericordiae University Hospital is a teaching hospital associated with University College Dublin, but University College Dublin will not be paying the publication costs for this study. The publication costs will be paid by Manchester Metropolitan University under a publication agreement with PLOS as one of our authors, Professor Gillian Yeowell, is employed as a professor in the university.
2. One of our authors, Dervilla Danaher, retired from the Mater Misericordiae University Hospital on 23/1/2025 and no longer has access to her work email. She has consented to being contacted by PLOS One on her personal email dervillad@gmail.com.

Kind regards,

Darragh Carolan
